# Implicit and Explicit Illusory Correlation as a Function of Political Ideology

**DOI:** 10.1371/journal.pone.0096312

**Published:** 2014-05-12

**Authors:** Luciana Carraro, Paolo Negri, Luigi Castelli, Massimiliano Pastore

**Affiliations:** 1 Department of Developmental and Socialization Psychology, University of Padova, Padova, Italy; 2 Department of General Psychology, University of Padova, Padova, Italy; University of Bologna, Italy

## Abstract

Research has demonstrated that people who embrace different ideological orientations often show differences at the level of basic cognitive processes. For instance, conservatives (vs. liberals) display an automatic selective attention for negative (vs. positive) stimuli, and tend to more easily form illusory correlations between negative information and minority groups. In the present work, we further explored this latter effect by examining whether it only involves the formation of explicit attitudes or it extends to implicit attitudes. To this end, following the typical illusory correlation paradigm, participants were presented with members of two numerically different groups (majority and minority) each performing either a positive or negative behaviour. Negative behaviors were relatively infrequent, and the proportion of positive and negative behaviors within each group was the same. Next, explicit and implicit (i.e., IAT-measured) attitudes were assessed. Results showed that conservatives (vs. liberals) displayed stronger explicit as well as implicit illusory correlations effects, forming more negative attitudes toward the minority (vs. majority) group at both the explicit and implicit level.

## Introduction

In our everyday life, we are endlessly confronted with examples of how liberals and conservatives embrace different views of the world and prioritize different goals and values [1]. Recent research has shown that these differences are skin deep and involve personality traits (e.g. [2], [3]), facial features [4], cognitive styles [5], and even brain structures [6]. In particular, conservatives, as compared to liberals, appear to more carefully process negative than positive environmental information. In a key study, Oxley and colleagues [7] showed that the exposure to threatening stimuli (e.g., a bloody face) gave rise to higher changes in skin conductance among conservative participants, as compared to liberal participants. Similarly, basic attention processes differ as a function of political ideology. For instance, it has been shown that negative information automatically attracts the attention of conservatives [8], [9]. In experimental tasks, such as a Dot-Probe Task, conservatives preferentially shift their attention towards threatening rather than positive stimuli [8], indicating that they are personally far more relevant than positive stimuli.

The asymmetrical processing of positive and negative information has important implications for how liberals and conservatives form impressions about novel social objects. In an intriguing study, Shook and Fazio [10] demonstrated that conservatives are more likely to display a learning asymmetry, namely a tendency to learn negative items relatively better than positive items, suggesting that the former might actually weigh more on the impressions formed by conservatives. People embracing conservative views of the world are also more susceptible to conditioning with negative stimuli than conditioning with positive stimuli [11], and this result points again to the presence of ideology-based asymmetries in the formation of social attitudes.

In a different line of research, Castelli and Carraro [12] explored impression formation processes relying on an illusory correlation paradigm. Illusory correlation is the tendency to misperceive the covariation between two events, and, more specifically, the tendency to believe that two relatively infrequent events are associated with each other even though no such association is actually present ([13], for a review see [14]). In the seminal study by Hamilton and Gifford [13], participants were presented with negative and positive actions performed by various members of two social groups (for instance, Group A and Group B). In the classic paradigm, one group is numerally larger (i.e., the majority group) as compared to the other (i.e., the minority group). In addition, the relative frequency of positive and negative actions is also manipulated, so that positive actions are more frequent than negative actions. Importantly, however, the ratio between negative and positive behaviors is exactly the same within the two groups, and thus there is no factual basis for evaluating one group as better than the other; nonetheless, participants tend to perceive a (illusory) correlation between the two infrequent information (e.g., between being a member of the minority group and performing negative behaviors). Moreover, Castelli and Carraro [12] found that illusory correlation effects were modulated by participants' political ideology, so that conservatives were even more likely to associate the minority group with negative behaviors and the authors reasoned that this was due to the increased distinctiveness of negative information in the case of conservative participants.

In the present study we first attempted to replicate the effects of political conservatism on illusory correlation and, most notably, explored whether the effects are limited to self-reported attitudes or extend to implicit attitudes. To our knowledge, only one experimental work has so far investigated the formation of both implicit and explicit attitudes within an illusory correlation paradigm [15]. Across two studies, the well-established illusory correlation effects were found on the explicit attitude measures but no effect emerged on the implicit attitude measure (i.e., an IAT) [16]. The authors interpreted the findings as the outcome of two largely independent learning processes (i.e., belief-based and contingency-based, respectively), so that implicit evaluations end up being unbiased, reflecting the actual contingencies in the observed information. Other lines of research, however, would lead one to expect illusory correlation effects to emerge on implicit attitudes as well [17]. According to Kruschke's attention theory of category learning [18]–[20], people first learn the attributes of the category that, within one context, is relatively larger than the other, and then learn the properties of the second category, giving more emphasis to what enables to distinguish it from the larger category. This implies that in the typical illusory correlation paradigm, participants first develop an impression about Group A and, because positive behaviors are indeed more frequent than negative behaviors, the final impression will be largely positive. Afterwards, the impression of Group B is formed and negative behaviors will attract the attention because they allow to better distinguish Group B from Group A. After the association between Group A and positive features is established, in relation to Group B there would be an attention-shifting mechanism that would lead perceivers to focus on the group-attribute pairs that maximize intergroup differentiation and, in that specific case, the focus would be on the association between Group B and negative behaviors. An important assumption of the model is that these processes occur while participants read the behavioral information about the two groups [17], [18]. Participants rapidly identify that one group is numerically larger than the other – at least when the ratio between the two groups is high - and set in motion the processes described above that imply a preliminary focus on the majority group followed by an attention-shifting mechanism toward the features of the minority group that allow one to maximize intergroup differentiation. As said, these processes occur at encoding during the initial learning phase, while participants are presented with the behavioral information, and therefore they could potentially impact onto the formation of specific associations between Group A (vs. Group B) and positive (vs. negative) features. In other words, the greater attention that is devoted to the instances in which members of Group B perform negative behaviors might strengthen the associative link between such group and negative features. According to this rationale, illusory correlation effects should be detected on both explicit and implicit attitude measures. In the present study we will thus further explore how illusory correlation may affect the formation of explicit and implicit attitudes, as well as how political ideology may modulate the effects. Overall, we expect that conservatives will display stronger illusory correlation effects on both implicit and explicit attitudes.

## Method

### Participants

One-hundred and twenty-three students (95 females) aged between 18 and 35 years (*M* = 19.56, *SD* = 2.19) participated in the study in exchange of course credits. The experiment was conducted in accordance with the guidelines laid down in the Declaration of Helsinki; participants provided written consent and all procedures were approved by the Human Subjects Ethics Committee at the School of Psychology of the University of Padova. Data are available upon request.

### Attitude induction procedure

Participants were seated in front of a computer screen and introduced to a memory task. It was explained that their task was to pay close attention to a series of sentences because they would be later asked questions about the content of such sentences. Next, participants were actually shown 39 sentences each describing a behavior performed by either a member of Group A or B. Group A was larger than Group B (26 vs. 13 exemplars). Sentences were either positive (27 sentences; e.g., “Jim, who belongs to Group A/B, has given way to sit to an elderly lady.”) or negative (12 sentences; e.g., “James, who belongs to Group A/B, usually tells many lies.”), and the ratio between negative and positive behaviors was identical within the two groups (i.e., 0.44). Thus, members of Group A performed 18 positive and 8 negative behaviors, whereas members of Group B performed 9 positive and 4 negative behaviors. Sentences were presented one after the other in a random order and each of them remained visible for 7 seconds. Between one sentence and the following there was an interval of 1 second. Subsequently both implicit and explicit attitudes toward the two groups were measured in a counterbalanced order.

### Dependent measures

#### Implicit attitudes

As for the implicit attitudes, an Implicit Association Test (i.e., IAT) [16] was used. The IAT assessed the automatic association between Group A vs. Group B (as stimuli we used sentences like “Jim belonging to Group A/B”) and positive (pleasure, happiness, liking, wonderful, joy, love) vs. negative words (pain, horrible, tremendous, disaster, bad, death). The IAT consisted of 5 blocks. In the first block (learning block with 20 trials) participants were asked to categorize positive and negative words. Subsequently, in the second learning block (20 trials) participants were asked to categorize members of Groups A and members of Group B. Then, in the third block (i.e., a critical block, 40 trials) participants were asked to categorize positive (vs. negative) words and members of Group A (vs. Group B) using the same response key. In the fourth block (learning block with 20 trials) they were asked to categorize members of Groups A vs. members of Group B, as in the second block, but the meaning of the response keys was reversed. Finally, in the last block (i.e., a critical block, 40 trials) they were asked to categorize positive (vs. negative) words and members of Group B (vs. Group A) using the same response key. The order of the two critical blocks (i.e., 3 and 5), as well as the order of the second and the fourth block, was counterbalanced across participants.

#### Explicit attitudes

First of all, data from six participants were not included in the analyses because they did not complete the questionnaire. As for the explicit attitudes toward the two groups, participants were initially required to evaluate the two groups along 11 traits (funny, irritable, intelligent, stupid, willing, sociable, brilliant, lazy, happy, unhappy, unpleasant). Responses had to be provided along 5-point Likert scales from 1 ( =  not at all) to 5 ( =  very much). Then, participants were asked to evaluate each group along 6 semantic differentials (unsociable/sociable, irresponsible/responsible, likeable/unlikeable, unpleasant/pleasant, hostile/friendly, unpopular/popular) with a 8-point scale. Subsequently, participants were provided with the full list of behaviors and asked to indicate the group membership of the actor of each behavior. This cued recall task enabled to assess how many positive and negative behaviors were attributed to the two groups. Next, a conceptually similar measure was administered and participants were asked to estimate how many behaviors had been performed by members of Group A and how many of them were negative. Identical questions were administered in relation to Group B.

#### Political Ideology

Political ideology was assessed in a separate moment through an online questionnaire. Participants were asked to report their level of agreement (from 1  =  “not at all” to 7  =  “very much”) with 12 different topics (such as reduction of immigration, medically assisted procreation, homosexual marriage, use of arms for personal defense, adoption by homosexual couples; after appropriate rescaling assigning higher values to conservative ideologies, α = .76, *M* = 3.15, *SD* = .87).

## Results

### Illusory correlation

#### Implicit attitudes

As for the IAT (split half reliability α = .88), a D score was calculated for each participant following the indications provided by Greenwald and colleagues [21]. As indicated by the authors [21] the D-IAT index is quite similar to the effect-size measure, Cohen's d. Positive scores indicated an illusory correlation between Group B and negative behaviors and therefore a more positive evaluation of Group A as compared to Group B. The index (*D-IAT*  = 1.01, *SD* = .60) emerged to be significantly higher than zero, *t*(121)  = 18.38, *p*<.001. The effect was not affected by the order in which the implicit and explicit measures were administered, *t*(120) = −.66, *p* = .51, suggesting that the more negative implicit attitude towards Group B did not emerge exclusively after the request to report one's explicit attitude.

#### Explicit attitudes

After appropriate rescaling (i.e., high scores indicate more positive evaluations), the mean evaluation of Group A (α = .82) and Group B (α = .84) along the 11 traits were separately calculated. A t-test showed more negative attitudes toward Group B (*M* = 3.11, *SD* = .52) than Group A (*M* = 3.70, *SD* = .45), *t*(116)  = 8.30, *p*<.001, *d = 1.21*. As for the semantic differential, values were recoded in such a way that positive scores indicate more positive evaluations and then a mean score was calculated for Group A (α = .88) and for Group B (α = .70). Group A was found to receive more positive evaluations (M = 5.10, SD  = .98) as compared to Group B (M = 4.20, SD = .84), *t*(116)  = 6.48, *p*<.001, *d = 0.99*. As for the cue recall task, a phi coefficient correlation was computed for each participant [12], [13] in such a way that positive values indicated an illusory association between Group B (vs. Group A) and negative (vs. positive) behaviors. The observed value was indeed positive (*M* = .21, *SD*  = .42) and significantly higher than zero, *t*(116)  = 5.45, *p*<.001, *d = 0.92*. As for the estimation of the frequency of negative behaviors within each group, we calculated the perceived proportion of negative behaviors given the overall number of behaviors attributed to the group. As expected, the proportion was higher in relation to Group B (*M* = .59, *SD* = .19) than Group A (*M* = .39, *SD* = .19), *t*(116)  = 6.69, *p*<.001, *d = 1.05*. The relative order of administration of the implicit vs. explicit measures had no impact on explicit attitudes (all t*s* <1.34, p*s* >.18).

#### Correlations between implicit and explicit attitudes

For each explicit measure a difference score was calculated so that positive scores always indicated more positive evaluations toward Group A as compared to Group B, thus demonstrating an illusory correlation between positivity (vs. negativity) and Group A (vs. Group B). Overall, implicit attitudes (i.e., IAT scores) were positively correlated with all explicit measures. More specifically, the individual implicit attitude was significantly correlated with the responses to the first explicit measure (evaluative ratings task), *r*(116)  = .21, *p* = .03, and with responses to the second explicit measure (semantic differentials), *r* (116)  = .20, *p* = .03, whereas the correlation with the responses in the cue recall task, *r*(116)  = .17, *p* = .06, and with the estimation of the frequency of negative behaviors within each group only approached the conventional level of significance, *r*(116)  = .17, *p* = .06.

### Illusory correlation as a function of ideology

In order to analyze the role of political ideology in the illusory correlations effects, a series of regression analyses were performed. As for implicit attitudes, a positive relation was found, β = .21, *t*(115)  = 2.28, *p* = .024, indicating that conservatives were more likely to display automatic associations between Group B (vs. Group A) members and negative (vs. positive) words as compared to liberals.

From further regression analyses political ideology emerged as a significant predictor of the differential evaluation of the two groups both along the 11 traits, β = .27, *t*(110)  = 2.99, *p* = .004, and along the 6 semantic differentials, β = .23, *t*(110)  = 2.50, *p* = .014. Also the phi coefficient calculated in relation to the cued recall task was positively associated to political ideology, β = .23, *t*(110)  = 2.45, *p* = .016. Finally, from responses to the last explicit measure a difference between the proportion of negative behaviors attributed to Group B and Group A was calculated. Again, political ideology emerged as a significant predictor, β = .20, *t*(110)  = 2.12, *p* = .036. Overall, the regression analyses consistently showed that stronger illusory correlation effects emerged for conservatives as compared to liberals both at the level of explicit attitudes as well as at the level of implicit attitudes.

The pattern of results described here has been confirmed also by a series of mixed models including “Group Type” (majority vs. minority; *D in the models below*) in the first model (i.e., m1 below). Next, in the second model (i.e., m2), we added the political ideology (*X in the models below)*, and then in the third and last model (i.e., m3) we added the interaction between Group Type (majority vs. minority) and political ideology. The equations of the models are reported below:
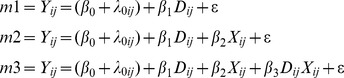

*where i* ( =  participants); *j* ( =  group); * Y* indicates the dependent variable (Explicit evaluation/Semantic differential/Ratio); *D* indicates the dummy variable associated to “Group Type” (0  =  Group A, majority group; 1  =  Group B, minority group); *X* stands for political ideology; *β* are the coefficients of the fixed part of the model; *λ* represents the random effects of participants.

For all the variables, the model including the interaction between type of group and political ideology (m3) provided the best fit to the data. Moreover, these analyses indicated that political ideology was related both to the evaluation of the majority group and to the evaluation of the minority group. Results are reported in Table 1.

**Table 1:** Results emerged by a series of mixed-models.

	Model	df	AIC	BIC	Chi-square	p-value	Bayes-Factor
**Explicit Evaluation**	Model 1	4	334.77	348.59			
	Model 2	5	328.74	345.76	8.03	*p*<.01	
	Model 3	6	320.25	340.66	10.50	*p*<.001	12.78
**Semantic Differential**	Model 1	4	629.23	643.05			
	Model 2	5	608.03	625.04	23.20	*p*<.001	
	Model 3	6	601.82	622.23	8.21	*p*<.001	4.07
**Ratio**	Model 1	4	−105.63	−91.81			
	Model 2	5	−88.75	−71.74	0.001	*p* = 1	
	Model 3	6	−93	−72.56	6.23	*p* = .01	1.51

AIC and BIC indicate Akaike Information Criterion [33] and Bayesian Information Criterion [34] respectively. Bayes Factor is approximated by formula 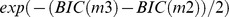 [35].

Finally, in order to explore whether illusory correlation can be detected among liberals, we performed a series of additional analyses centering the predictor of political ideology at one standard deviation above and below the ideology mean, and then we examined whether the intercept was significant in order to understand whether a bias occurs for both conservatives and liberals, respectively. As for the IAT, results indicated that a bias emerged both for conservatives, β  = 1.13, *t*(114)  = 14.42, *p*<.001, and liberals, β = .88, *t*(114)  = 11.03, *p*<.001. As for the cued recall task, results from a similar analysis indicated an illusory correlation bias both for conservatives, β = .31, *t*(109)  = 5.31, *p*<.001, and for liberals, β = .11, *t*(109)  = 1.98, *p* = .05. This pattern of results emerged also for the other explicit variables, namely the explicit evaluation of the two groups (difference score) [β = .81, *t*(109)  = 7.76, *p*<.001, for conservatives, β = .38, *t*(109)  = 3.78, *p*<.001 for liberals], the semantic differentials (difference score) [β = 1.26, *t*(109)  = 6.07, *p*<.001 for conservatives, β = 0.54, *t*(109)  = 2.70, *p* = .008 for liberals] and the estimation of the ratio of negative behaviors performed by the two groups [β = .26, *t*(109)  = 5.92, *p*<.001,for conservatives, β = .13, *t*(109)  = 3.09, *p* = .003 for liberals]. Current results seem to indicate that an implicit and explicit illusory correlation bias emerged both in the case of liberals and in the case of conservatives, although remarkably stronger in the latter case.

## Discussion

Nowadays there is a renewed interest in the study of the likely differences that characterize conservatives vs. liberals [22], [23]. Literature has largely documented how political orientation and ideology are not only related to different views of the world, but also to more deep cognitive and automatic processes. For instance, conservatives appear to be more sensitive to negative stimuli as compared to positive stimuli [7]–[11], and negative information immediately captures the automatic attention of conservatives [8]. This attentional asymmetry between people who support different ideologies may, at least partially, explain why conservatives often develop more negative impressions about social minorities. Indeed, conservatives appear to weigh negative information more than positive information and this leads to even more biased impressions in the classic illusory correlation paradigm [12]. In the present study we replicated this effect showing that the explicit evaluations and the memory performance of conservatives were more likely affected by an illusory correlation bias. Even though there was no factual basis for considering the minority group as less positive than the majority group, people in general, and conservatives in particular, ended up with such an inaccurate perception. Notably, similar effects were also observed on implicit attitudes, as assessed through the IAT. Conservatives, indeed, showed a stronger tendency to automatically associate the minority group (vs. majority group) to negative features (vs. positive features) suggesting that ideological individual differences can shape, in an illusory correlation paradigm, both explicit and implicit attitudes. This is very much relevant to the extent that explicit and implicit attitudes are related to the regulation of different types of behaviors. Whereas explicit attitudes mainly predict deliberate behavior, implicit attitudes are more strongly related to spontaneous behavior [24]–[27]. For instance, explicit attitudes are correlated with the content of verbal behaviors and implicit attitudes are better predictors of nonverbal and uncontrolled behaviors that are displayed while interacting with the attitude object. In addition, implicit attitudes are more likely to predict behaviors under specific circumstances [28]–[29], such as reduced cognitive capacity and ego depletion, that temporarily weaken central executive control. Implicit and explicit attitudes also differ in relation to how they change over time. Indeed implicit attitudes (relative to explicit attitudes) change more slowly and are more impermeable to disconfirming information that is subsequently encountered [30]. Taken together, it appears that conservatives, once confronted with a minority group that displays relatively infrequent negative behaviors with the same probability of a majority group, develop widespread negative attitudes that prepare them to negatively react toward such minority group.

The findings from the present study are also relevant at a more general level for the understanding of illusory correlation phenomena. Until now, to the best of our knowledge, only one study [15] has investigated illusory correlation effects on both explicit and implicit attitudes concluding that two different processes are involved, leading to distinct attitudes: biased explicit attitudes but unbiased implicit attitudes. In sharp contrast, we here found that the illusory correlation bias was not restricted to explicit evaluations but it also emerged when automatic associations were assessed. Indeed, from responses in the IAT [16], [21], participants revealed more negative implicit attitudes toward the minority group as compared to the majority group. Moreover, implicit and explicit attitudes appeared to be somehow correlated to each other, whereas no order effects emerged to be significant. These results are in line both with the “strategic explanation” proposed by Fiedler [31], [32] and with the “attention theory of category learning” proposed by Kruschke [18]. Indeed, according to the “strategic explanation” [31], [32] while reading evaluative information about the two groups, each positive or negative action performed by a member of a group reinforces the association between that specific group and positive vs. negative evaluations. This rationale may explain both implicit and explicit illusory correlation effects. In addition, the same effects may also be explained according to Kruschke's theory [18]. The theory states that people initially form an impression (in this case positive) about the majority group and then give more emphasis to the features (in this case negative) that distinguish one group from the other. Importantly, this process would occur while participants are engaged into reading the behavioral information about the two groups [17], [18], thus enabling to form biased explicit as well as implicit attitudes. However, further studies will have to better investigate why in some cases illusory correlation effects emerge at both the implicit and explicit level whereas in some other cases illusory correlation effects are restricted to explicit attitudes [15]. The methodology used in the present study differs from that employed by Ratliff and Nosek on several respect, and therefore one may only speculate about the origin of the obtained different findings. It is noteworthy that previous studies [15] made use of fictitious names to designate both the groups and group members and this may have hindered impression formation processes. Interestingly, the effects on explicit measures were also remarkably weaker in Ratcliff and Nosek's studies as compared to those observed here, suggesting that the methodology used in their studies was less likely to trigger illusory correlation phenomena overall, and therefore reducing the likelihood of detecting any effect at the implicit level. Moreover, because of the difference between the present findings and those reported by Ratcliff and Nosek, we performed an additional study (N = 86, F = 68, age *M* = 19.20, *SD* = .87) using the very same material and procedure adopted in the present study, with only one difference, namely the name of the two groups (Group Blue and Group Yellow instead of Group A and Group B). The key effect on the implicit measure was replicated and, indeed, a significant implicit preference for the majority group over the minority group emerged (*D-IAT*  = 1.17, *SD*  = .70; *t*(85)  = 2.11, *p* = .030).

To conclude, the current results confirm and extend previous findings [12] indicating that political orientation can actually impact onto the formation of both explicit and implicit attitudes in an illusory correlation paradigm. Indeed, conservatives developed more negative explicit and implicit attitudes toward the minority group as compared to the majority one. This result is important not only from a theoretical standpoint but also for its practical implications, suggesting that basic cognitive processes, such as those involved in the illusory correlation bias, may contribute to the more likely emergence of negative attitudes toward minorities among conservatives.

## References

[1] Caprara GV, Schwartz SH, Capanna C, Vecchione M, Barbaranelli C (2006) Personality and politics: Values, traits, and political choice. Polit Psychol 27: 1–28.

[2] Adorno TW, Frenkel-Brunswick E, Levinson DJ, Sanford RN (1950) The authoritarian personality. New York: Harper & Row.

[3] Caprara GV, Zimbardo PG (2004) Personalizing politics: A congruency model of political preference. Am Psychol 59: 581–594. 15491254 10.1037/0003-066X.59.7.581

[4] Rule NO, Ambady N (2010) Democrats and Republicans can be differentiated from their faces. PLoSONE 5: e8733. 10.1371/journal.pone.0008733 PMC2807452 20090906

[5] Jost JT, Glaser J, Kruglanski AW, Sulloway FJ (2003) Political conservatism as motivated social cognition. Psychol Bull 129: 339–375. 12784934 10.1037/0033-2909.129.3.339

[6] Kanai R, Feilden T, Firth C, Rees G (2011) Political orientations are correlated with brain structure in young adults. Curr Biol 21: 677–680. 21474316 10.1016/j.cub.2011.03.017 PMC3092984

[7] Oxley DR, Smith KB, Alford JR, Hibbing MV, Miller JL, et al (2008) Political attitudes vary with physiological traits. Science 321: 1667–1670. 18801995 10.1126/science.1157627

[8] Carraro L, Castelli L, Macchiella C (2011) The automatic conservative: Ideology-based attentional asymmetries in the processing of valenced information. PLoSONE 6: e26456. 10.1371/journal.pone.0026456 PMC3212508 22096486

[9] Dodd MD, Balzer A, Jacobs CM, Gruszczynski MW, Smith KB, et al (2012) The political left rolls with the good and the political right confronts the bad: connecting physiology and cognition to preferences. Philos T Roy Soc B 367: 1589. 10.1098/rstb.2011.0268 PMC3260844 22271780

[10] Shook NJ, Fazio RH (2009) Political ideology, exploration of novel stimuli, and attitude formation. J Exp Soc Psychol 45: 995–998.

[11] Shook NJ, Clay R (2011) Valence asymmetry in attitude formation: A correlate of political ideology. Soc Psychol Pers Science 2: 650–655.

[12] Castelli L, Carraro L (2011) Ideology is related to basic cognitive processes involved in attitude formation. J Exp Soc Psychol 47: 1013–1016.

[13] Hamilton DL, Gifford RK (1976) Illusory correlation in intergroup perception: A cognitive basis of stereotypic judgments. J Exp Soc Psychol 12: 392–407.

[14] Stroessner SJ, Placks JE (2001) Illusory correlation and stereotype formation: Tracing the arc of research over a quarter century. In Moskowitz GB, editor. Cognitive social psychology: The Princeton symposium on the legacy and future of social cognition Mahwah, NJ: Erlbaum. pp. 247–259.

[15] Ratliff KA, Nosek BA (2010) Creating distinct implicit and explicit attitudes with an illusory correlation paradigm. J Exp Soc Psychol 46: 721–728.

[16] Greenwald AG, McGhee DE, Schwartz JLK (1998) Measuring individual differences in implicit cognition: The implicit association test. J Pers Soc Psychol 74: 1464–1480. 9654756 10.1037//0022-3514.74.6.1464

[17] Sherman JW, Kruschke JK, Sherman SJ, Percy EJ, Petrocelli JV, et al (2009) Attentional pocesses in stereotype formation: A common model for category accentuation and illusory correlation. J Pers Soc Psychol 96: 305–323. 19159134 10.1037/a0013778

[18] Kruschke JK (1996) Base rates in category learning. J Exp Psychol Learn 22: 3–26. 10.1037//0278-7393.22.1.3 8648289

[19] Kruschke JK (2001) The inverse base-rate effect is not explained by eliminative inference. J Exp Psychol Learn 27: 1385–1400. 10.1037//0278-7393.27.6.1385 11713874

[20] Kruschke JK (2003) Attention in learning. Curr Dir in Psychol Sci 12: 171–175.

[21] Greenwald AG, Nosek BA, Banaji MR (2003) Understanding and using the Implicit Association Test: An improved scoring algorithm. J Pers Soc Psychol 85: 481–481. 10.1037/0022-3514.85.2.197 12916565

[22] Jost JT (2006) The end of the end of ideology. Am Psychol 61: 651–670. 17032067 10.1037/0003-066X.61.7.651

[23] Amodio DM, Jost JT, Master SL, Yee CM (2007) Neurocognitive correlates of liberalism and conservatism. Nat Neurosci 10: 1246–1247. 17828253 10.1038/nn1979

[24] Dovidio JF, Kawakami K, Gaertner SL (2002) Implicit and explicit prejudice and interracial interaction. J Pers Soc Psychol 82: 62–68. 11811635 10.1037//0022-3514.82.1.62

[25] Hofmann W, Gschwendner T, Castelli L, Schmitt M (2008) Available control resources moderate the relative impact of the impulsive and reflective system on prejudiced behaviour. Group Process Interg 11: 69–87.

[26] McConnell AR, Leibold JM (2001) Relations among the Implicit Association Test, discriminatory behavior, and explicit measures of racial attitudes. J Exp Soc Psychol 37: 435–442.

[27] Asendorpf JB, Banse R, Mücke D (2002) Double dissociation between implicit and explicit personality self-concept: The case of shy behavior. J Pers Soc Psychol 83: 380–393. 12150235

[28] Hofmann W, Rauch W, Gawronski B (2007) And deplete us not into temptation: Automatic attitudes, dietary restraint, and self-regulatory resources as determinants of eating behavior. J Exp Soc Psychol 43: 497–504.

[29] Friese M, Hofmann W, Schmitt M (2008) When and why do implicit measures predict behavior? Empirical evidence for the moderating role of opportunity, motivation, and process reliance. Eur Rev Soc Psychol 19: 285–338.

[30] Rydell RJ, McConnell AR (2006) Understanding implicit and explicit attitude change: A systems of reasoning analysis. J Pers Soc Psychol 91: 995–1008. 17144760 10.1037/0022-3514.91.6.995

[31] Fiedler K (1991) The tricky nature of skewed frequency tables: An information loss account of distinctiveness-based illusory correlations. J Pers Soc Psychol 60: 24–36.

[32] Fiedler K (2000) Illusory correlations: A simple associative algorithm provides a convergent account of seemingly divergent paradigms. Rev Gen Psychol 4: 25–58.

[33] Akaike H (1974) A new look at the statistical model identification. IEEE T Automat Contr 19: 716–723.

[34] Schwarz GE (1978) Estimating the dimension of a model. Ann Stat 6: 461–464.

[35] Raftery AE (1995) Bayesian model selection in social research. Sociol Methodol 25: 111–163.

